# Os Vesalianum Pedis in a Young Adult: A Case Report and Literature Review

**DOI:** 10.7759/cureus.14896

**Published:** 2021-05-07

**Authors:** Vasileios K Mousafeiris, Ioannis Papaioannou, Nektaria Kalyva, Christine Arachoviti, Thomas Repantis

**Affiliations:** 1 Orthopedics, General Hospital of Patras "Agios Andreas", Patras, GRC; 2 Pediatrics, General University Hospital of Patras, Patras, GRC

**Keywords:** os vesalianum pedis, ankle injury, foot injury, lateral foot pain, iselin’s disease, jones fracture, metatarsal avulsion fracture

## Abstract

Os vesalianum pedis is a rare accessory foot ossicle. It is usually asymptomatic, however, it can be an infrequent cause of lateral foot pain. We present the case of a 19-year-old healthy male with lateral foot pain after an inversion-type injury. Initial X-rays were mistaken for fracture of the fifth metatarsal, however, a high index of suspicion for the presence of the os vesalianum led us to perform imaging of the contralateral foot; a mirror image with contralateral os vesalianum was revealed. The patient was treated conservatively and had an excellent outcome. In the context of trauma, os vesalianum must be differentiated from other causes of lateral foot pain, such as Iselin’s disease, avulsion fracture of the fifth metatarsal, Jones fracture, and others. Os vesalianum pedis is characterized as a rounded ossicle, with smooth edges, surrounded by cortical bone. Prompt diagnosis is essential as it changes the management.

## Introduction

Os vesalianum pedis (OVP) is one of the many accessory bones which can be found in the human foot. These accessory bones of the foot are considered to be skeletal variations. The reported incidence of the most common accessory bones in adult feet is: accessory navicular (11.7%), os peroneum (4.7%), os trigonum (2.3%), os supranaviculare (1.6%), os supratalare (0.2%), and os intermetatarseum (0.2%) [1]. OVP is a rare accessory ossicle of the foot with incidence ranging between 0.1% and 5.9% [1-3].

It is mostly asymptomatic and incidentally recognized. However, it may sometimes cause lateral foot pain. To our knowledge, only 11 cases of symptomatic OVP have been reported in the literature.

We, hereby, present a rare case of symptomatic OVP and only the third one that was treated conservatively. We emphasize careful clinical and radiological evaluation of every patient with lateral foot pain in similar cases, to avoid wrong diagnosis and subsequent overtreatment. 

## Case presentation

A 19-year-old healthy male was admitted to the emergency department of our hospital complaining of lateral foot pain. He reported minor trauma, with possible inversion-type injury of the lateral foot a few hours before the ER admission. The pain was localized to the fifth metatarsal base at the attachment of the peroneus brevis tendon. The patient did not report lateral foot pain previously. On physical examination, slight prominence was seen at the fifth metatarsal base that was tender on palpation. Inversion and plantar flexion of the foot was painful. The ankle joint range of motion was normal without instability findings. The neurovascular examination was also normal. The Foot and Ankle Disability Index (FADI) score was 54 points (52%) [4].

**Figure 1:**
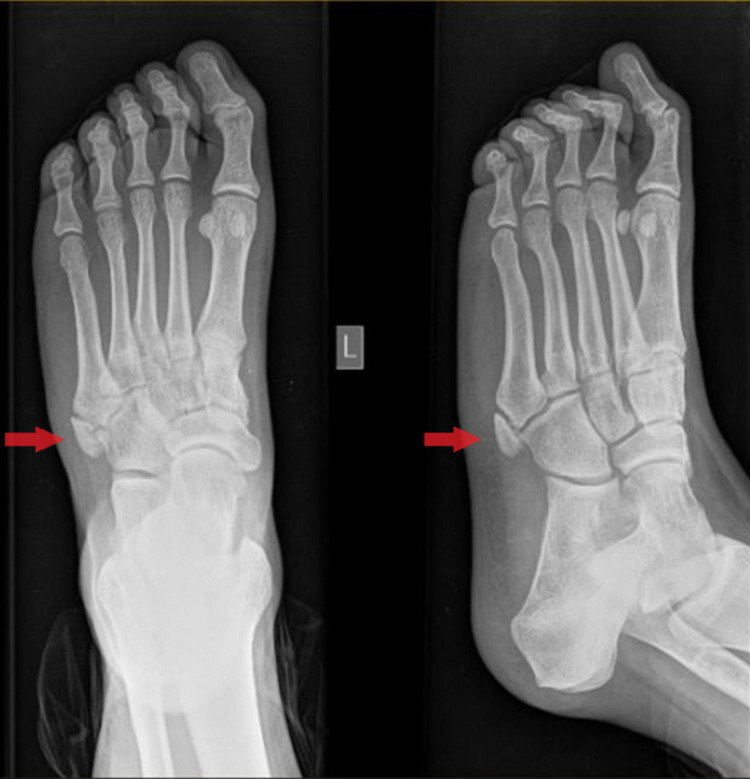
Plain radiograph (AP and lateral oblique) of the left foot reveals the presence of the os vesalianum pedis articulating with both the base of the fifth metatarsal and the cuboid bone (red arrow). AP: anteroposterior

**Figure 2:**
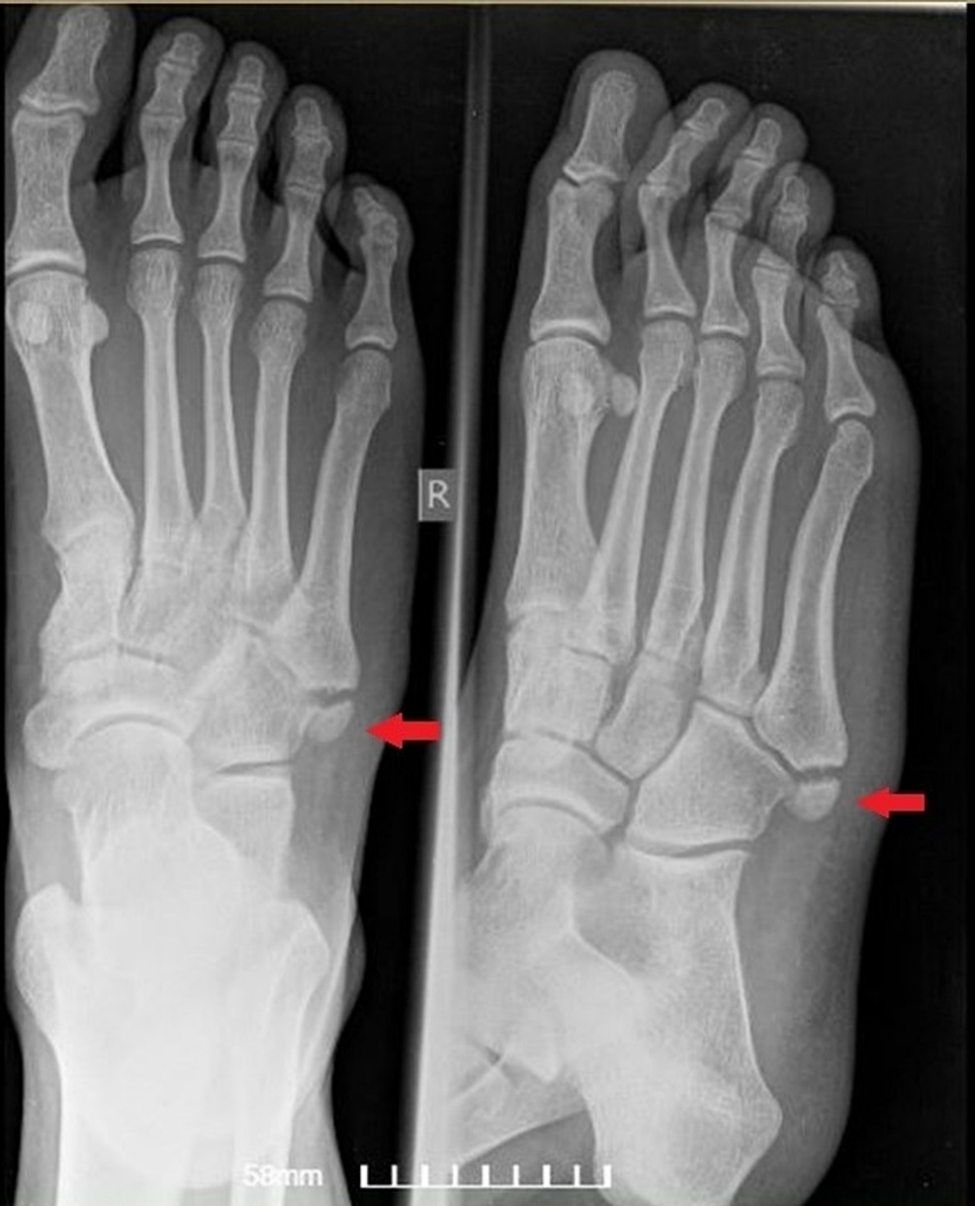
Plain radiograph (AP and lateral oblique) of the contralateral foot reveals the presence of a similar os vesalianum pedis (“mirror image”) (red arrow). AP: anteroposterior

Plain radiographic examination (anteroposterior {AP} and lateral oblique radiograph) of the left foot showed a bean-shaped oval bone fragment at the fifth metatarsal base (Figure 1). This initial radiographic imaging was perceived as an acute fracture of the fifth metatarsal base. However, a high clinical index of suspicion for the presence of OVP led us to perform imaging of the contralateral (healthy) foot. Imaging of the contralateral foot revealed an identical bean-shaped bony fragment, as seen in the injured foot (mirror image) (Figure 2). Furthermore, re-evaluation of the initial foot radiographs suggested an ossicle at the base of the fifth metatarsal, rather than a fracture. The ossicle was separated from the metatarsal by a radiolucent line of constant width, with a well-corticated edge (Figure 1). Therefore, the diagnosis of OVP was established. The patient was treated conservatively, with rest, ice, leg elevation, and compression along with mild pain medication, and was discharged home.

**Figure 3:**
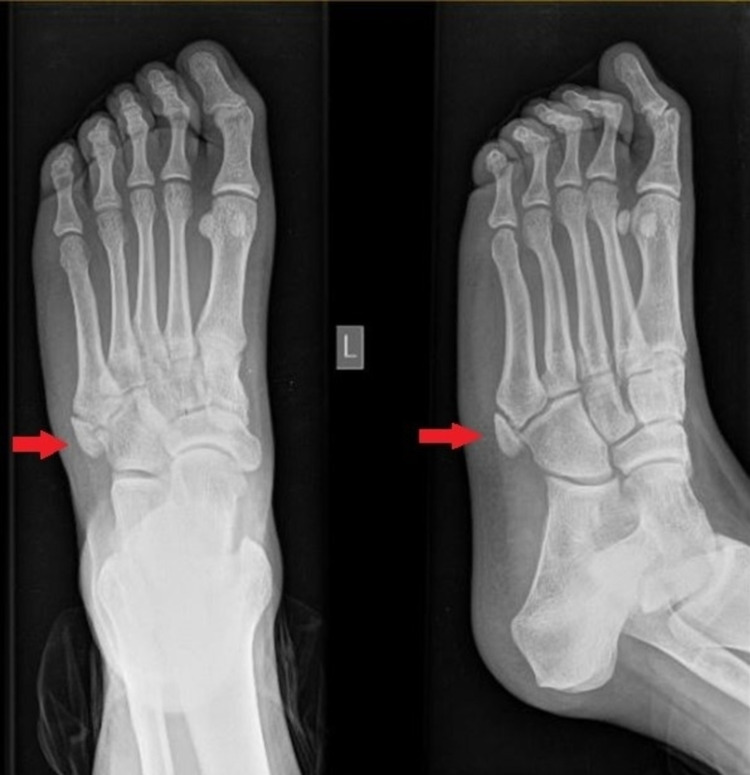
Plain radiograph (AP and lateral oblique) of the left foot (injured) during the first follow-up confirms the presence of the os vesalianum pedis articulating with both the base of the fifth metatarsal and the cuboid bone (red arrow). The image is identical to the initial X-ray performed during ED admission. AP: anteroposterior

On the follow-up examination 20 days later, the patient had complete remittance of the lateral foot pain and edema. The Foot and Ankle Disability Index (FADI) score was 104 points (100%) [4]. Radiographic imaging of the injured foot was identical to the initial one (Figure 3). In further follow-up at six weeks after injury, the patient was again symptom-free. The Foot and Ankle Disability Index (FADI) score was again 104 points (100%), so the patient was discharged from our care.

## Discussion

The OVP is a rare accessory ossicle of the foot, with incidence ranging between 0.1% and 5.9% [1-3]. We present a rare case of OVP that became symptomatic after an acute ankle sprain. To our knowledge, this is only the third case of a symptomatic OVP that is treated conservatively. Although most of the reported cases have been treated surgically, the pertinent literature is not devoid of cases treated conservatively [5,6]. Our patient was clearly asymptomatic until an inversion injury of the left foot occurred.

Two similar OVP cases have been presented to our knowledge. They both reported young males admitted to the ED with an acute ankle sprain and lateral foot pain. Radiography revealed an accessory ossicle. In the case presented by Kose, the OVP was initially perceived as a fracture of the base of the fifth metatarsal, like in our case, and below-the-knee plaster was applied. On follow-up, re-evaluation of the initial radiographs confirmed the diagnosis of the OVP, and the patient was treated conservatively with ankle sprain management recommendations [5]. In the case presented by Boya et al., clinical suspicion for the presence of OVP and radiography of the contralateral (healthy) foot revealed the presence of the OVP bilaterally, like in our case [6]. The diagnosis was confirmed by a CT scan; the patient was again treated conservatively. The follow-up for both patients was excellent in subsequent follow-ups, as in our case.

The exact origin of the OVP is not known, however, few theories exist. One theory proposes that the OVP is a persistent apophysis [7]. During skeletal development, at the age of 10 years for girls and 12 years for boys, an apophyseal line at the base of the fifth metatarsal is seen in X-rays. Fusion of this apophysis occurs usually two years later (from the age of 12 years for girls and 14 years for boys) [8]. Another theory suggests that the OVP is caused by a pseudarthrosis of a fifth metatarsal tuberosity avulsion fracture [9]. However, the most widely accepted theory is that the OVP is a true sesamoid bone as dictated by the presence of its articular cartilage [1].

The OVP is an infrequent cause of lateral foot pain. In fact, the presence of OVP does not mean that it will be necessarily symptomatic and will cause foot pain and disability [5]. The differential diagnosis of lateral foot pain in a young patient includes a variety of clinical entities other than the OVP, namely Iselin’s disease, avulsion fracture of the fifth metatarsal base, Jones fracture, metatarsal stress fracture, and os peroneum [5,10,11]. The differential diagnosis also includes nonunion of a tuberosity fracture, an ununited apophysis, or an ossifying apophysis of the fifth metatarsal base [8].

Iselin’s disease is a traction apophysitis of the fifth metatarsal base and should be taken into consideration, especially in the pediatric population and young adolescents [8]. The cause of Iselin’s disease is probably the repetitive microtrauma that occurs from the traction of the peroneus brevis tendon on the unfused apophysis, hence it is an overuse injury [8,11]. Lateral foot pain is the most common symptom and is aggravated during sports, particularly running. Its characteristic is that when the apophysis fuses (at about the age of 12 years for girls and 14 years for boys), then the pain subsides. Radiographic imaging in Iselin’s disease demonstrates apophyseal fragmentation and irregularity. Radiographs may be negative early in the course of the disease [12]. MRI findings precede the radiographic epiphyseal fragmentation and are thus useful in the early detection of the disease [13]. Comparing OVP with Iselin’s disease, age can be considered a useful screening tool, as Iselin’s disease usually presents in young adults and worsens with sports activities. On the other hand, the presence of the OVP exists beyond adulthood. Furthermore, symptomatic Iselin’s disease-causing lateral foot pain has no history of trauma, contrary to the OVP which becomes symptomatic usually after an ankle sprain or inversion forces of the foot [11-14].

In the context of trauma, OVP can be misdiagnosed as a fifth metatarsal avulsion fracture, Jones fracture, or fifth metatarsal stress fracture [5,10]. If so, patients are treated with unnecessary immobilization [5,14]. However, OVP has characteristics typical of an accessory bone that help for the identification and prompt diagnosis. It is usually separated from the base of the fifth metatarsal by a synchondrosis line that follows an oblique course. The ossicle has also a rounded shape with smooth edges and is surrounded by cortical bone. However, the most prominent characteristic of the OVP is the articulation with the cuboid bone [1-3,6]. The above features are not present in the fractures of the fifth metatarsal base, as the fracture line is sharp and the bony fragment lacks cortication [5]. Furthermore, in all types of fractures, including avulsion fractures, Jones fracture, and stress fractures, the orientation of the fracture line and the apophyseal line is quite different and almost always perpendicular to each other. More specifically, the apophysis appears as a longitudinal line parallel to the long axis of the fifth metatarsal, whereas avulsion fractures usually have a transverse orientation [5,13]. Radiographically, a Jones fracture is a transverse fracture at the junction of the diaphysis and metaphysis that does not extend distal to the fourth and fifth intrametatarsal articulation. Avulsion fractures are located in the proximal pole of the fifth metatarsal. A stress fracture of the proximal fifth metatarsal is a diaphyseal fracture that is located at the proximal 1.5 cm of the shaft, as a result of chronic repetitive trauma. Stress fractures are more distal and in the transverse plane on plain radiographs [11,14].

Os peroneum is also a bean-shaped sesamoid bone that must be differentiated from OVP. It is usually found within the distal end of the peroneus longus tendon. Although it articulates with the cuboid bone, like OVP, the os peroneum is smaller [15]. Along with clinical suspicion, imaging plays an important role in the diagnosis of the OVP. As OVP is in most cases bilateral, both feet X-rays are important for the initial screening. MRI can also be performed; however, it may be inconclusive [16]. Bone scintigraphy has been also used to further assess the cause of OVP [14].

Initial treatment of symptomatic OVP should start with rest, shoe inserts, stretching exercises, non-steroidal anti-inflammatory drugs (NSAIDs), limited weight-bearing, and/or casting [6,9,17-20]. In cases resistant to conservative treatment, surgical options should be considered. Surgical options involve excision of the symptomatic OVP without disruption of the peroneus brevis tendon insertion, as well as osteosynthesis and bone grafting; both treatments have been reported with good functional outcomes [9,11,14,17-20]. However, it is important to ensure that the OVP is the cause of the lateral foot pain in order to avoid maltreatment. 

## Conclusions

It is important to be aware of the possible presence of OVP as an infrequent cause of lateral foot pain in order to avoid overtreatment. OVP can be perceived as a possible fracture of the fifth metatarsal base and a high index of suspicion is needed in order to reach the correct diagnosis. Differential diagnosis includes, apart from fractures of the fifth metatarsal (avulsion fracture, Jones fracture, stress fracture), Iselin’s disease, and other accessory ossicles. A lateral oblique radiograph is the best method for demonstrating accessory ossicles and their articulations. Treatment is initially conservative, while surgery is reserved for refractory cases.
